# Squeezable Hydrogel Microparticles for Single Extracellular Vesicle Protein Profiling

**DOI:** 10.1002/smll.202407809

**Published:** 2024-10-29

**Authors:** Yoon Ho Roh, Renee‐Tyler T. Morales, Emily Huynh, Uday Chintapula, David E. Reynolds, Renis J. Agosto‐Nieves, Daniel Oh, Akari J. Seiner, Jianhua Lim, Christopher B. Rodell, Jina Ko

**Affiliations:** ^1^ Department of Pathology and Laboratory Medicine University of Pennsylvania Philadelphia PA 19104 USA; ^2^ Department of Bioengineering University of Pennsylvania Philadelphia PA 19104 USA; ^3^ School of Biomedical Engineering Science and Health Systems Drexel University Philadelphia PA 19104 USA

**Keywords:** digital assays, extracellular vesicles, multiplexing, rolling circle amplification, squeezable hydrogel

## Abstract

Extracellular vesicles (EVs) are promising for molecular diagnostics, but current analyses are limited by the rarity and compositional heterogeneity of EV protein expression. Therefore, single EV profiling methods require high sensitivity, multiplexing, and throughput to address these issues. Here a single EV analysis technique that utilizes squeezable methacrylated hyaluronic acid hydrogel microparticles (MHPs) is described as a scaffold to immobilize EVs and perform an integrated rolling circle amplification (RCA) assay for an ultra‐sensitive and multiplex analysis of single EV proteins. EVs are prepared into MHPs in a high‐throughput manner with droplet microfluidics and optimally labeled with antibody‐oligonucleotide conjugates in MHPs without steric limitations. By designing MHPs with high compressibility, single EV protein signals are amplified as RCA products that can be aligned on the same plane by physically squeezing MHPs and visualized with low magnification. This method provides a simple and scalable single EV imaging analysis pipeline for identifying multiplex marker expression patterns from single EVs. For validation, the single EV heterogeneity of highly expressed cancer cell markers is profiled across different cancer cell lines. These findings exemplify squeezable MHPs as a robust platform with high sensitivity, multiplexing, and scalability for resolving single EV heterogeneity and advancing molecular assay technologies.

## Graphical abstract

Methacrylated Hyaluronic Acid Hydrogel Microparticles (MHPs) are a scaffold for encapsulating EVs and performing a rolling circle amplification (RCA) assay for a multiplex protein profiling of single EVs without steric limitations. With high compressibility, MHPs can be squeezed for single RCA products of antibody‐labeled EVs to be aligned and imaged on the same plane. Therefore, a simple, scalable analysis pipeline is presented to uniquely profile rare protein expression patterns and resolve single EV heterogeneity.

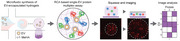

## 1 Introduction

Extracellular vesicles (EVs) are actively shed by cells in both healthy and pathological states.^[^ ^1^ ^]^ They are increasingly recognized as potential circulating sources of biomarkers.^[^ ^2^ ^]^ To date, areas of precision medicine such as oncology increasingly rely on biopsied tumor tissue. However, sampling beyond the initial tissue biopsy is limited. Therefore, circulating biomarkers (“liquid biopsy”) are ideal for analysis. Notably, EVs exhibit high stability for protecting molecular cargo and are present in various biofluids including saliva, urine, and blood.^[^ ^3^ ^]^ EVs shuttle diverse molecules including proteins and nucleic acids that reflect the composition of their parent cells and studies have suggested that EV markers are superior in sensitivity to cell‐free DNA (cfDNA) for cancer diagnosis.^[^ ^4^ ^]^ However, the molecular heterogeneity of EVs complicates their utility.

Identifying disease‐specific EVs, especially in the earliest stages of cancer development, is notoriously difficult because of the overwhelming background of EVs shed by healthy cells and their scarce presence.^[^ ^5^ ^]^ The stochastic nature of EV biogenesis, the rarity of disease‐specific EVs, the abundance of background EVs, and the heterogeneity of molecular markers expressed in these nanometer particles render bulk analysis challenging.^[^ ^6^ ^]^ Hence, high‐throughput single EV or “digital” profiling methods can address these technical gaps to resolve the molecular heterogeneity of EVs and advance diagnostic assays. A number of single EV analysis methods have been developed, including fluorescence microscopy of EVs on glass,^[^ ^7^ ^]^ small particle flow cytometry,^[^ ^8^ ^]^ and digital protein platforms.^[^ ^9^ ^]^ However, these techniques are restricted by their own limitations, which include the size detection limits and cost of high‐resolution optics for flow cytometry and fluorescence microscopy; the need for large EV sample amount for flow cytometry; the low signal sensitivity of traditional EV immunofluorescence staining; and the Poisson distribution and multiplexing restrictions of digital ELISA.

To address the limitations of current techniques, hydrogel microparticles are an ideal reaction vessel to perform ultra‐sensitive and multiplex measurements of single EVs. Hydrogel microparticles have been widely used in bioassays^[^ ^10^ ^]^ to load and profile a broad range of targets including cells,^[^ ^11^ ^]^ nanoparticles,^[^ ^12^ ^]^ and molecules (*e.g*. protein, RNA, and DNA).^[^ ^13^ ^]^ Their porous mesh‐like structure allows for optimal molecular interactions in a 3D space, exhibiting solution‐like kinetics^[^ ^14^ ^]^ suitable for profiling EVs without steric limitations. Also, the non‐fouling nature of hydrogel materials inhibits nonspecific molecules binding in complex matrices,^[^ ^15^ ^]^ increasing the sensitivity of rare protein detection of EVs. Although digital ELISA platforms such as droplets^[^ ^9d^ ^]^ and microwells^[^ ^16^ ^]^ have shown high sensitivity, the Poisson distribution is a significant limitation as the number of compartments to isolate a single EV dictates the sensitivity of the assay with ≈10% containing a single EV. To overcome this, localized in situ amplification strategies such as hybridization chain reaction (HCR) and rolling circle amplification (RCA) can be employed to locally amplify protein signals from single EVs as it has been done with hydrogel‐based analyses from EV lysates,^[^ ^17^ ^]^ cells,^[^ ^18^ ^]^ and tissues.^[^ ^19^ ^]^ As a result, integrating hydrogel microparticles with localized in situ amplification can enable simultaneous orthogonal amplification for a multiplexed fluorescent imaging of single EVs without the need for single particle compartmentalization.

Herein, we demonstrate the use of squeezable hydrogel microparticles as a scaffold to immobilize EVs and conduct an integrated RCA assay for an ultra‐sensitive and multiplex protein analysis of single EVs. This involves the high throughput generation of uniform droplets consisting of methacrylated hyaluronic acid (MeHA) polymer precursor and bulk EVs with a flow‐focusing microfluidic droplet generator. EVs are physically arrested within hydrogel microparticles by photopolymerization. Consequently, captured EVs are labeled with DNA barcoded antibodies (Ab‐DNA) and multiplex protein signals are amplified by incorporating RCA into the hydrogel microparticle assay without Poisson and steric limitations. We validated that MeHA hydrogel microparticles (MHPs) exhibit high compressibility upon physical squeezing, which can align RCA products in a single plane to uniquely enable imaging without the need for high‐magnification z‐stack imaging. As a result, image acquisition is cost‐effective, simple, and fast; and EV analysis is high throughout as a single image contains >10^3^ EVs. An automated pipeline was developed to describe the presence/absence and co‐expression of protein markers for single EVs from different cancer cell lines. For validation, we profiled the protein expression patterns of highly expressed cancer cell markers among single EVs from different cancer lines and elucidated their heterogeneity. As a result, this method bypasses the Poisson distribution and multiplexing limits of digital ELISA platforms; enhances sensitivity by enabling larger surface area of interactions with epitopes; and provides a scaffolding for conducting RCA steps to enhance visualization of rare protein signals. Consequently, we present a simple, robust, and scalable platform that can enable the identification of rare EV subpopulations with diagnostic potential and advance the development of molecular assay technologies.

## 2 Results and Discussion

### 2.1 Synthesis and Characterization of MHPs

**Figure 1:**
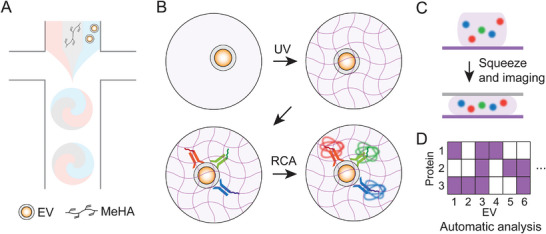
Overview of the multiplex single EV analysis using MeHA hydrogel microparticles (MHPs). A) Droplets including EVs and precursor, composed of MeHA and photoinitiator, are fabricated by microfluidic technique. B) MHPs were prepared by exposing UV to droplets, which physically capture EVs. The synthesized MHPs were incubated with antibody‐DNA (Ab‐DNA) followed by rolling circle amplification (RCA) to amplify protein markers from single EVs. C) MHPs were squeezed to align the RCA dots on the same plane for the imaging. D) Customized image analysis software automatically analyzes the presence or absence and colocalization of protein markers from single EVs.

**Figure 2:**
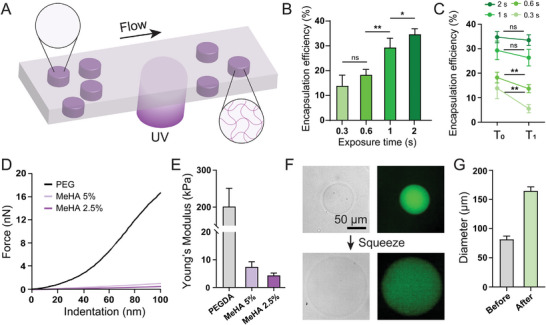
Preparation and characterization of MHPs. A) MHPs were produced by photocuring droplets in the polymerization chamber. B) The UV exposure time was varied to evaluate the encapsulation efficiency of MHPs using 100 nm fluorescent beads as model EVs. C) Assessment of remaining fluorescent beads in MHPs before (T0) and after (T1) the assay procedure according to the different UV exposure times. Error bars indicate the standard deviation from five MHPs D) Force‐indentation curves and E) Young's modulus for hydrogels synthesized by PEG and two different concentrations of MeHA. F) Micrographs and G) diameter of MHPs before and after squeezing. Error bars indicate the standard deviation from ten particles. (^*^ *p* < 0.05, ^**^ *p* < 0.01)

We first fabricated MHPs by developing a flow‐focusing microfluidic droplet generator and a UV polymerization chamber for synthesizing and crosslinking MeHA hydrogel droplets for EV immobilization and downstream RCA‐based protein analysis (**Figure** **1**). MeHA was synthesized by methacrylation of hyaluronic acid through esterification with methacrylic anhydride, and the degree of substitution was quantified by 1H NMR (Figure S1, Supporting Information).^[^ ^20^ ^]^ A prepolymer solution containing MeHA, EV, and a photoinitiator was injected as a dispersed phase into a droplet generator, which includes a curved structure after the pinch‐off region for enhanced mixing efficiency (Figure S2, Supporting Information). Subsequently, droplets were reinjected into the polymerization chamber to be crosslinked into the hydrogels by continuous flow photocuring (**Figure** **2**A). The height of the polymerization chamber (50 µm) was set to be smaller than the diameter of the droplets to make spheroid‐like structures with flat tops and bottoms for accurate measurement of modulus and uniform compression. The optimal concentration of MeHA was found to be 2.5% (w/v), which can reduce the Young's modulus for squeezing while maintaining a stable, solid structure.^[^ ^21^ ^]^ We confirmed that the MHPs are in the hydrogel state and performed Environmental SEM to visualize the morphology of MHPs (Figure S3, Supporting Information). It should be noted that the mesh size of the MHPs was large enough for allowing full penetration of antibodies while preventing the diffusion of ≈100 nm lipid nanoparticles. As a result, physically captured EVs remain immobile while other reagents for downstream protein signal amplification can be successfully diffused into MHPs (Figure S4, Supporting Information).

To assess the capture efficiency of EVs within the MHPs, 100 nm fluorescent beads were utilized as a model for EVs and tested under various crosslinking conditionsby varying UV exposure times (Figure 2B). The capture efficiency was observed to be higher when the MHPs were synthesized under prolonged UV exposure times, due to the formation of denser networks within the MHPs. Also, the remaining beads within the MHPs were validated after the completion of the assay procedures, including shaking and incubation at elevated temperatures (Figure 2C). A comparison of the results obtained from the initial (T_0_) and final (T_1_) assay procedures revealed that MHPs synthesized under low UV exposure times exhibited a significantly higher degree of leakage (60.1% for 0.3 s) than those synthesized under high UV exposure times (3.6% for 2 s). Combining encapsulation efficiency and leakage during the assay procedures, 5.7%, 13.8%, 26.4%, and 34.7% of beads remained for MHPs synthesized at 0.3, 0.6, 1, 2 seconds of UV exposure respectively, and we chose 1 s as an optimal UV exposure time to enhance the permeability of MHPs (data not shown).

We further characterized the mechanical properties of MHPs using atomic force microscopy (AFM). For comparison against MHPs, PEG‐based hydrogel microparticles (PHPs), which have been frequently used for bioassay purposes,^[^ ^13^, ^22^ ^]^ were fabricated. Nanoindentation of MHPs and PHPs revealed a significant difference in the modulus of the two groups, and a decrease in MeHA concentration leads to a decrease in modulus (Figure 2D,E). Subsequently, the compressibility of MHPs and PHPs was evaluated by applying a force (≈960 µN) to the particles. As expected, MHPs demonstrated high compressibility (Figure 2F), whereas PHPs were unable to be compressed (Figure S5, Supporting Information). The diameter of MHPs increased by a factor of two, resulting in a reduction in height from 50 to 12.5 µm (Figure 2G). Note that increasing the UV exposure time during the MHP synthesis resulted in decreasing compressibility of MHPs (Figure S6, Supporting Information). The reduced height is comparable to the z‐axis resolution of the 20x objective lens, ranging from 5.2 to 8 µm, calculated by Abbe's diffraction formula when considering fluorophores from AF405 to Cy5. This indicates that MHPs can be imaged in a single plane without the need for z‐stack imaging.

### 2.2 Optimization of RCA

**Figure 3:**
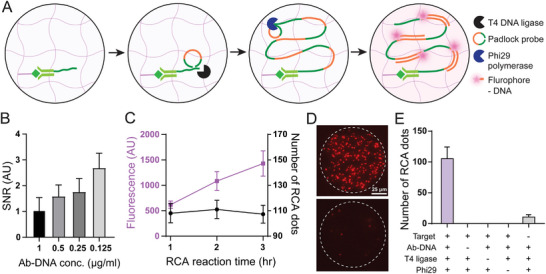
RCA‐based assay validation and optimization. A) Schematic of the RCA assay within the hydrogel networks. B) Optimization of Ab‐DNA concentration. The signal‐to‐noise ratio (SNR) was estimated as the ratio of RCA dots between targeted and control (no targeted protein) MHPs. Data are displayed as mean ± sd from five replicates. C) Optimization of RCA time. The fluorescence intensity of MHPs (purple) and the number of RCA dots (black) were measured during amplification. Error bars indicate the standard deviation from five MHPs. D) Fluorescence micrographs of targeted and control MHPs after RCA‐based assay. E) Assay validation. RCA dots were observed only when all assay components were included. Bars indicate the number of dots from five MHPs.

Due to its unique features, RCA has been widely adopted in molecular detection assays that are facilitated on solid support or complex mesh‐like structures such as hydrogel microparticles.^[^ ^17^, ^23^ ^]^ RCA products grow to a size of hundreds of nanometers to a few micrometers and remain anchored within the hydrogel networks, which allows a digital counting of individual RCA dots from MHPs.^[^ ^24^ ^]^ The workflow to perform the RCA‐based assay is represented as follows (**Figure** **3**A). The initial step involves the capture of target proteins by DNA‐conjugated antibodies. This is followed by the introduction of padlock probes (PLP), designed with arms that hybridize to DNA. After T4 DNA ligase is introduced to form a circular DNA template, phi29 polymerase is added to continuously synthesize long strands of DNA based on the sequence of the PLP. This long single‐stranded concatemer of DNA is tagged by multiple fluorophore‐labeled DNA probes, resulting in an amplification of the protein signal.

To optimize RCA using MHPs, we targeted epidermal growth factor receptor (EGFR) from the lysate of A431 cells, a cell line highly enriched with EGFR protein.^[^ ^25^ ^]^ Protein molecules from A431 cell lysate were loaded into the droplets during the fabrication process and conjugated to the remaining unreacted methacrylate groups via aza‐Michael addition reaction (Figure S7, Supporting Information).^[^ ^26^ ^]^ We first varied the concentration of antibody‐DNA (Ab‐DNA) conjugates, prepared by TCO/Tz chemistry (see Methods section for details), to determine the best signal‐to‐noise ratio (SNR)^[^ ^9b^ ^]^ and selected the Ab‐DNA concentration of 0.125 µg mL^−1^, which maximized the SNR (Figure 3B). In addition, the optimal reaction time for RCA was determined (Figure 3C). To verify the consistency of the reaction without the formation of background RCA dots throughout the RCA process, the number of RCA dots was measured at each time point (1, 2, 3 h). It was confirmed that the number of RCA dots remained consistent, while the fluorescence intensity from MHPs increased with reaction time. The optimal reaction time was set to be 3 h, at which point the fluorescence intensity of the RCA dots was sufficiently strong to be analyzed in the imaging conditions. Also, the formation of RCA products was validated by the presence or absence of essential RCA components as variables. We demonstrated that the RCA dots were successfully generated only when all assay components were present, whereas missing assay components were unable to generate RCA dots (Figure 3D,E).

### 2.3 Characterization of EVs Using MHPs

**Figure 4:**
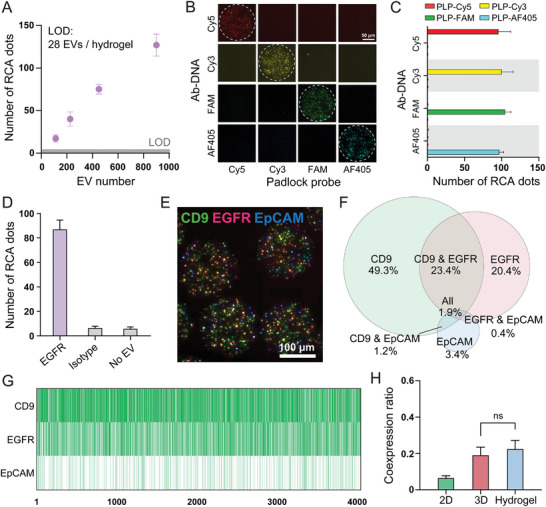
Characterization of EVs using MHPs. A) A calibration curve with varying amounts of A431 EVs. Error bars are standard deviations from seven MHPs. B,C) RCA probe sets specificity. Fluorescence micrographs and bar graphs demonstrated high specificity of the designed probe sets. Error bars are standard deviations from seven MHPs. D) Evaluation of assay specificity. Bars indicate the number of RCA dots from A431 EVs from ten MHPs. E) Multiplex analysis of A431 EVs. Fluorescence micrographs of MHPs after 3‐plex assay. F) Venn diagram showed percentage of single, double and triple positive EVs among the tested markers. G) Mapping of single EV. Each row represents a single biomarker, and each column represents a single EV. H) Comparison of co‐expression ratio from surface (2D), solution (3D), and hydrogel assay by analyzing CD9 and EGFR from A431 EVs.

After optimizing RCA using MHPs, our technology was applied to the analysis of EVs. EVs derived from A431 cells were selected as a model system, with the EGFR protein marker serving as the target for validation. Cryo Electron Microscopy was performed to visualize EVs to rule out contamination of other cellular components (Figure S8, Supporting Information). A series of titration studies demonstrated that the limit of detection (LOD) was 28 EVs/hydrogel (**Figure** **4**A). The LOD was 6 times lower than that of flow cytometry which is used as a gold standard method for single EV profiling.^[^ ^27^ ^]^ Subsequently, four distinct probe sets (Ab‐DNA barcode/PLP/fluorophore‐labeled DNA) were designed to expand the multiplexing capacity of our technology.^[^ ^28^ ^]^ The specificity of the probe set was assessed by modifying the anti‐EGFR antibody with four distinct barcodes and reacting with each PLP/fluorophore‐labeled DNA set (Figure 4B,C). It should be noted that the cell lysate was used for the probe set specificity test to avoid the heterogeneity of signals associated with EVs. The results confirmed two important factors for multiplexing: 1) high specificity with negligible nonspecific signal; and 2) similar reactivity between probe sets. To evaluate the specificity of our technology, a control experiment was performed to compare the number of RCA dots obtained from no EV control and isotype control (anti‐IgG isotype antibody) to those obtained with target‐specific antibodies (anti‐EGFR antibody) (Figure 4D). The number of RCA dots from both the no EV control and the isotype control was considerably lower than that of the positive control, indicating that the RCA dots are generated in a highly specific manner. We also confirmed that the UV irradiation during the synthesis of MHPs did not affect the structural integrity or functionality of surface proteins on EV (Figure S9, Supporting Information). Furthermore, we confirmed with a non‐cancerous EV control derived from healthy human embryonic kidney (HEK293) cells that the specificity of our Ab‐DNA conjugates are truly specific to cancer cell‐specific EVs. As expected, both HEK293 and A431 cell line derived EVs express CD9, a universal exosome marker, but HEK293 expresses minimal or negligible EGFR and EpCAM cancer protein markers (Figure S10, Supporting Information).

We then demonstrated the multiplex detection of A431 EVs by targeting three surface markers of EVs: EGFR, epithelial cell adhesion molecule (EpCAM), and cluster of differentiation 9 (CD9). EGFR and EpCAM were chosen as abundant cancer markers for A431 and CD9 was chosen as one of the universal EV markers, which is known as the most prevalent marker among the tetraspanins.^[^ ^7b^ ^]^ To analyze different single‐positive, double‐positive, and triple‐positive EV subtypes, we developed a customized analysis software based on CellProfiler.^[^ ^29^ ^]^ The customized pipeline enables segmentation and counting of RCA dots from different fluorescence channels, which leads to the automation of the analysis process (Figure S11, Supporting Information). Note that the single RCA dot was considered as a single EV, regardless of the size of the RCA dot. To account for background RCA dots, the same number of EVs from fetal bovine serum (FBS) was used as a control. As the background dots of FBS from each marker are not overlapped, the number of background dots was subtracted solely from the single‐positive dots. Applying this strategy, we analyzed the distribution of three markers in A431 EVs, and revealed that 73.1%, 25%, and 1.9% of EVs were single‐positive, double‐positive, and triple‐positive, respectively (Figure 4E,F). Among single positive EVs, 67.4% was from CD9, while 28% and 4.6% were from EGFR and EpCAM, respectively. Most of the double‐positive EVs originated from CD9 and EGFR colocalization, which is the combination of an abundant protein marker from A431 and one of the EV markers. Additionally, a comprehensive single EV map was constructed across three markers from more than 4 000 EVs (Figure 4G).

Until now, glass or gold surfaces have been frequently used as a substrate for single EV analysis methods.^[^ ^7^, ^30^ ^]^ Although these methods demonstrated their utility in single EV detection, we speculated that the analysis from the surface‐based EV capture assay might underestimate the populations of single EVs due to the steric restrictions of the 2D surface.^[^ ^31^ ^]^ Therefore, we tested the co‐expression ratio of CD9 and EGFR from A431 EVs using a 2D surface assay and small particle flow cytometry (Figure 4H; Figure S12, Supporting Information). Nano‐flow cytometry was employed to explore an ideal 3D solution‐based assay in which no steric restrictions were applied during the assay. Interestingly, there was a significant difference in co‐expression ratio between the 2D surface‐based assay and 3D solution‐based assay with the results of the hydrogel‐based assay being similar to the 3D solution‐based assay. This is because the hydrogel provides optimal molecular interactions in 3D space, thus minimizing steric hindrance during the assay. By comparing co‐expression ratios, we demonstrated that hydrogel‐based single EV detection can be more accurate than surface‐based assays in terms of identifying marker expression.

### 2.4 Profiling Cancer Cell EV Protein Marker Heterogeneity Using MHPs

**Figure 5:**
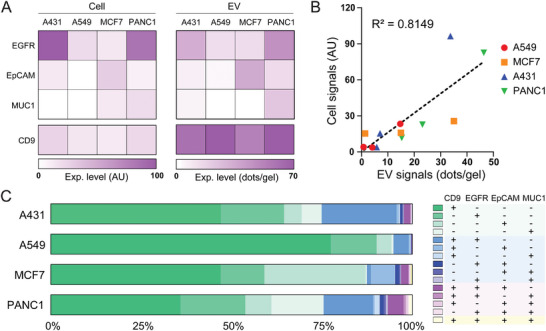
Multiplex single EV profiling reveals heterogeneity of pan‐cancer marker expression among EVs shed by different cancer cell lines. A) Molecular protein expression of pan‐cancer markers (EGFR, EpCAM, and MUC1) and EV tetraspanin marker CD9 was characterized on parent cells and EVs derived from 4 cancer cell lines (A431, A549, MCF7, and PANC1). B) Correlation of cancer protein marker expression between EVs and respective parental cell lines. C) Analysis of biomarker concurrence (right; legend of protein marker combinations) across single EVs obtained from all four cell lines.

A number of highly expressed cancer markers have been identified for different cancers, but the use of a single marker is limited due to insufficient sensitivity or specificity. Following preliminary validation of the specificity and multiplexing, we proceeded to perform a 4‐plex analysis of single EVs among other cancer cell lines: A431 (epidermal), A549 (lung), MCF7 (breast) and PANC1 (pancreatic). To first calibrate and validate the MHP assay, we investigated the expression of cancer markers EGFR, EpCAM, and MUC1, as well as EV marker CD9, between parent cells and EVs by measuring fluorescence intensity from cell immunostaining and counting the number of RCA dots per hydrogel from the MHP assay, respectively. As reported in other literature, the whole cell protein expression patterns are as follows: both A431 and A549 cells highly express EGFR; PANC1 cells highly express both EGFR and MUC1; and MCF7 cells highly express EpCAM (**Figure** **5**A).^[^ ^25^, ^32^ ^]^ Furthermore, it was found that CD9 expression was highly enriched in EVs compared to whole cells, which is expected as it is a universal EV marker. As a result, the correlation analysis of cancer markers between parent cells and EVs excluded CD9. Each cell line's single EV counts reflect similar cancer protein expression profiles from whole cells with our data showing a positive correlation (R^2^ = 0.815) (Figure 5A,B). In addition to constructing comprehensive single EV maps that illustrate the heterogeneity of protein marker presence/absence for each cancer line (Figure S13, Supporting Information), we quantified the distribution of protein marker co‐expression for different marker combinations (Figure 5C). As expected, EV CD9 counts were relatively high in abundance (35% or higher) since tetraspanin markers such as CD9 should be constitutively expressed among EVs. Interestingly, EVs from A431 (17%), A549 (13%), MCF7 (12%), and PANC1 (18%) cell lines express EGFR only to a similar degree. There was a higher presence of co‐expressed EGFR and CD9 with A431 (21%) and PANC1 (14%) EVs, consistent with whole cell EGFR expression. Remarkably, MCF7 EVs express EpCAM with (6%) and without CD9 (28%) consistent with MCF7 cell expression. Meanwhile, most PANC1 EVs express MUC1 with (14%) or without CD9 (14%) which is in alignment with PANC1 cell expression. It was determined that less than 1% of EVs from different cancer lines express all markers. Despite their recognition as cancer markers, EGFR, EpCAM, and MUC1 were revealed by four different cancer line‐derived EVs to be expressed at different expression levels as expected of single EV protein heterogeneity and supports the necessity of ultra‐sensitive detection.

To demonstrate that the degree of multiplexing is not restricted by spectral overlap, we validated that the MHP assay is compatible with cyclic imaging. This was demonstrated with CD9 and EGFR signals, such that fluorophore‐labeled DNA following the first round of hybridization could be stripped from RCA products by formamide‐based denaturation within 10 min (Figure S14, Supporting Information). As a result, future studies can incorporate iterative rounds of fluorophore‐labeled DNA hybridization/stripping, enabling a higher order of multiplexing for single EV analysis. Altogether, we believe our findings exemplify our squeezable MHP assay's performance in sensitively detecting and profiling rare protein expression patterns of single EVs to resolve their molecular heterogeneity.

## 3 Conclusion

The promise of analyzing EVs for molecular diagnostics has produced significant interest, but current tools are limited by the rarity and compositional heterogeneity of EV protein patterns. There is a need for more accurate and sensitive techniques to validate the utility of EV biomarkers. The work presented here, a MHP assay integrated with RCA, enables a multiplex and ultra‐sensitive protein analysis of single EVs. The use of a squeezable hydrogel microparticle‐based assay addresses many limitations of existing single EV methods. These include overcoming the Poisson distribution and multiplexing limits of digital ELISA platforms; the sensitivity by enabling larger surface area of interactions with epitopes; and a scaffolding for conducting RCA steps to enhance visualization of rare protein signals. As a result, image acquisition is cost‐effective, simple, and fast; and EV analysis is high throughout as a single image contains >10^3^ EV and an automated pipeline was developed to describe the presence/absence and co‐expression of protein markers for single EVs from different cancer cell lines. This sensitive platform represents a promising path for defining rare single EV protein expression patterns for molecular assays.

While our technology has many advantages, there are some notable limitations of the present study. Despite the convenience of bulk mixing EVs, its low EV encapsulation efficiency limits its utility. However, future studies can address this limitation by modifying the HA backbone or using other polymers. For example, capture antibodies can be decorated on the backbone of HA through direct conjugation or molecular linkers (e.g., streptavidin‐biotin) to capture marker‐specific EVs. In addition, hydrogel particles can be synthesized using low‐molecular‐weight monomer (e.g., PEGDA 700), aiming for a smaller mesh size to enhance capture of EVs. To compensate for the reduced compressibility, the height of the hydrogel particles can be designed to match that of MHPs after squeezing. Although a 4‐plex analysis was performed, we have demonstrated that this platform is not constrained by spectral overlap. We validated that cyclic imaging can be integrated with RCA products to increase the degree of multiplexing possible. Lastly, while markers examined in this study are mainly expressed on the EV surface, this technique can be further expanded by labeling protein targets within EVs via semi‐permeabilization to discover new EV biomarker combinations. As a result, a comprehensive single EV analysis would allow the classification of rare EV subpopulations and enable organ‐of‐origin identification of cancer development from obtainable biofluids such as plasma. The development of a simple, robust, and scalable platform for resolving single EV heterogeneity will provide insights to studying EVs and addressing limitations in molecular diagnostics.^[^ ^20^, ^33^ ^]^

## Conflict of Interest

The authors declare no conflict of interest.

## Supporting information



Supporting Information

## Data Availability

The data that support the findings of this study are available from the corresponding author upon reasonable request.
