# Supplementary material for: Squeezable Hydrogel Microparticles for Single Extracellular Vesicle Protein Profiling
Source: Small. 2024 Oct 29;21(1):2407809. doi: 10.1002/smll.202407809 (PMC11707585; doi:10.1002/smll.202407809)
Supplement: Supplementary file 1 — Supporting Information [file SMLL-21-2407809-s001.docx]

**Squeezable Hydrogel Microparticles for Single Extracellular Vesicle Protein Profiling**

Yoon Ho Roh^[a]#^, Renee-Tyler T. Morales^[b]#^, Emily Huynh^[b]^, Uday Chintapula^[a]^, David E. Reynolds^[b]^, Renis J. Agosto Nieves^[b]^, Daniel Oh^[b]^, Akari J. Seiner^[c]^, Jianhua Lim^[b]^, Christopher B. Rodell^[c]^, Jina Ko^[a,b]^*

[a] YH Roh, U Chintapula, J Ko

Department of Pathology and Laboratory Medicine, University of Pennsylvania, Philadelphia, PA, 19104, USA

[b] RTT Morales, E Huynh, DE Reynolds. RJA Nieves, D Oh, J Lim, J Ko

Department of Bioengineering, University of Pennsylvania, Philadelphia, PA, 19104, USA

[c] AJ Seiner, CB Rodell

School of Biomedical Engineering, Science and Health Systems, Drexel University, Philadelphia, PA 19104, USA

^#^ These authors contributed equally to this work.

*Jina Ko, PhD

Department of Bioengineering

Department of Pathology and Laboratory Medicine 440 Curie Blvd, Philadelphia, PA, 19104

713-628-0283

[Jina.Ko@pennmedicine.upenn.edu](mailto:Jina.Ko@pennmedicine.upenn.edu)

**Methods**

**Fabrication of microfluidic device**

The device was first designed using a design software (AutoCAD, USA) and a 50 µm height SU-8 master mold was fabricated by a conventional photolithography process. To make the droplet generator, a Polydimethylsiloxane (PDMS; Corning, USA) mixture (10:1 base to curing agent ratio) was prepared, poured over the master mold, and cured (70 °C, overnight). The cured PDMS slab was then peeled off, cut into the desired size, and punched with holes (1 mm biopsy punch; Miltex, USA) to define fluidic ports. The bottom microfluidic substrate was prepared by coating a PDMS mixture onto glass slides and partially cured (60 °C, 2 h). The fluidic device was assembled by attaching the PDMS slab to the partially cured surface of the glass slides. The final device was cured (60 °C, overnight) to ensure leak-free bonding. To make the polymerization chamber, 1.6% (w/w) iron powder (Thermo Fisher Scientific, USA) was mixed into the PDMS mixture to prevent scattering of UV inside the channel.

**MeHA synthesis**

Hyaluronic acid (HA, 90kDa; Lifecore Biomedical) was methacrylated by esterification with methacrylic anhydride (MA).^[1]^ Briefly, HA (5.0 g) was dissolved in deionized (DI) water (500 mL) in a three-neck round bottom flask. The reaction was cooled on ice and the pH adjusted to 8.5 by the addition of 1N NaOH prior to the addition of MA (11.14 mL). The reaction was vigorously stirred, maintaining pH 7.5-8.5 by the addition of 1N NaOH for 3 h before allowing the solution to stir at room temperature overnight. Purification was performed by dialysis against DI water for 10 days (6-8kDa MWCO; Spectra/Por). The final product was frozen at -80°C, lyophilized, and characterized by 1H NMR (500 MHz; Varian Unity Inova). The degree of substitution was quantified by 1H NMR, determined to be 30%. From a single 1.0 g batch, aliquots of 3.8 mg of lyophilized MeHA were prepared for all MeHA hydrogel microparticle experiments.

**Hydrogel microparticle fabrication and squeezing**

A precursor solution was prepared by mixing 2.5% (w/v) MeHA dissolved in triethanolamine buffer (TEOA, 0.2 M, pH 8; Sigma Aldrich, USA) with 0.2% (w/v) Lithium Phenyl-2,4,6-trimethylbenzoylphosphinate (LAP; Takara, Japan) for MHP and 20% (v/v) of PEG700DA (Sigma Aldrich), 40% (v/v) of PEG600 (Sigma Aldrich) and 0.2% (w/v) LAP for PHP. Precursor was injected into the microfluidic device as a dispersed phase using a syringe pump (New Era Pump Systems Inc., USA) while HFE-7500 with 2% (w/w) fluorosurfactant (RAN biotechnologies, USA) was injected as a continuous phase. The generated droplets were reinjected to the polymerization chamber for gelation via UV exposure (10 mW/cm^2^). After the gelation, 1H,1H,2H,2H-perfluoro-1-octanol (Sigma Aldrich) was introduced to remove the surfactant and 1xPBST (1xPBS containing 0.05% (v/v) tween 20) was added for the phase transition from oil to aqueous phase. Finally, MHPs were washed three times in 1xPBST. To squeeze MHPs, a cover slip (22ⅹ22mm; Thermo Fisher Scientific) was placed on top of MHPs which were mounted on a glass slide.

**Characterization of mesh size**

For the lipid nanoparticle (LNP) synthesis, Dioleoyl-3-trimethylammonium propane (DOTAP), Dipalmitoylphosphatidylcholine (DPPC), Cholesterol and 1, 2-Distearoyl-sn-glycero-3-phosphoethanolamine-Poly(ethylene glycol) (DSPE-PEG2000, Avanti Research, USA) were dissolved at 10 mg/mL in ethanol lipids with a molar ratio of 40:40:10:10. Cy5 labeled PC lipids at 0.05% molar ratio were added to lipid mixture for fluorescent tagging of LNPs. 20mM HEPES buffer was used as aqueous phase for the formulation. Staggered herring bone micromixer was used to generate LNPs with a flow rate ratio of 1:3 (lipid in oil: aqueous) Then, LNPs were dialyzed against PBS to remove ethanol and characterized for their size and zeta potential using Dynamic Light Scattering.

For the diffusion study using Cy5-labeled LNPs, 50 µL of 2000 MeHA particles were incubated with 50 µL of 2.5 mg/mL Cy5-labeled LNPs for 120 min. For the diffusion study using antibodies, MHPs were conjugated with 6 mg/mL anti-EGFR (Cetuximab) through a thiol-ene reaction for 24hrs.^[2]^ Following three wash steps with 1xPBST, 10 µg/mL Goat anti-Human IgG-AF647 secondary antibody was introduced and incubated for 60 min. Before imaging, all MHPs were washed three times with 1xPBST.

**MHP capture efficiency measurements**

2% (v/v) Cy5-labeled 100 nm nanoparticles (Nanocs, USA) were encapsulated in MHPs with varying UV exposure times. Fluorescent images of MHPs were taken prior to the transfer to the aqueous phase. Afterwards, MHPs were washed three times in 1xPBST and fluorescent images were taken to measure the remaining nanoparticles. The capture efficiency was calculated by dividing the fluorescent intensity of the MHPs in the aqueous phase by the fluorescent intensity of the MHPs in the oil phase. To assess the leakage of nanoparticles during the assay procedure, MHPs were incubated at 25 °C for 1 hr and then incubated at 37 °C for 3 hr in a thermomixer (1500 rpm). Subsequently, fluorescent images of the MHPs were taken and the fluorescent intensity of the initial and final assay procedures was compared.

**Atomic Force Microscopy (AFM) measurements**

Young's modulus of MHPs and PHPs were measured using the Asylum AFM (Oxford Instruments, USA). MHPs mounted on a glass slide were fixed by Cell-Tak (Corning) and submerged in PBS for measurements. A spherical tip indenter, with a tip radius of 5 µm, was calibrated (spring constant 0.047 N/m) to probe the MHPs and PHPs to an indentation depth of 0.1-0.6 µm. Young’s modulus of the sample was obtained by fitting the force-indentation depth data to a Hertzian contact model.

$$F= \frac{4}{3}\frac{E}{(1-v^{2})}R^{\frac{1}{2}}\delta^{\frac{3}{2}}$$

where F is the force, E is the Young’s modulus, v is the Poisson’s ratio, R is the radius of the indenter tip and δ is indentation depth. Poisson ratio was set to 0.5.^[3]^

**EV isolation from cell culture**

A431, A549, MCF7, and PANC1 cell lines were purchased from the American Type Culture Collection. All cell lines were maintained in Dulbecco’s modified Eagle’s medium, supplemented with 10% fetal bovine serum, 100 IU of penicillin, and streptomycin (100 μg/ml). Cells were grown in a 150 mm cell culture dish and subsequently expanded to 12 dishes for EV isolation. After cells reached confluency, media was changed to exosome-depleted DMEM (5% exosome-depleted FBS, 1% penicillin). After 48 h from media exchange, the collected supernatant was spun at 400 g for 5 min and filtered with a 0.22 µm vacuum filter to remove cellular debris. The supernatant was centrifuged twice (Beckman Coulter, NC9146666) at 100,000 g for 70 min at 4 °C. The EV pellet was resuspended in PBS and further purified using size exclusion chromatography (70 nm qEV column, Izon science). From a 200 µL volume, 5 µL of 1011-1012 EVs/mL were stored as aliquots at −80 °C before use, and to eliminate batch heterogeneity for all EV analyses, they were sourced from the same isolated batch.

**EV characterization**

Following EV isolation, samples were characterized in two different ways. The protein concentration was measured using Qubit (Thermo Fisher) and the number of particles was calculated using nanoparticle tracking analysis (NTA). For Qubit, the protein assay kit (Thermo Fisher) was used and the company protocol was followed for measurement. For NTA, the measurement was carried out using the ZetaView PMX220 Twin instrument (Particle Metrix) at the University of Pennsylvania School of Veterinary Medicine Extracellular Vesicle Core. The analysis employed the following parameters: sensitivity of 65 and shutter of 100.

**EGFR detection from A431 cell lysate**

5.0E6 A431 cells were pelleted and treated with a 1X working concentration of protease inhibitor cocktail (Thermo Fisher, 78430) in 0.5 mL of RIPA lysis and extraction buffer (Thermo Fisher, 89900) on ice for 15 min. After incubation, the solution was centrifuged at 14,000 g for 15 min to pellet the cell debris. The supernatant was then transferred, and its protein concentration was assessed with Qubit. Protein was then stored at −80 °C until use.

**Antibodies**

Cetuximab (anti-EGFR antibody, Selleckchem, A2000), anti-EpCAM antibody (Bioxcell, BE0386), anti-MUC1 antibody (Biolegend, 355602), and anti-CD9 antibody (Biolegend, 312102). All antibodies were tested on positive cell lines and validated before use.

**Cell immunostaining**

All cell lines were individually passaged into an 8-chambered coverglass system (Cellvis, C8-1.5P) and grown to confluence prior to fixation with 4% PFA-PBS. Cells were blocked with 2% BSA-PBS to reduce background signals. Then, cells were successively treated with primary antibodies against EGFR, EpCAM, MUC1, and CD9 (5 µg/mL prepared in BSA-PBS) and Alexa Fluor 647 secondary antibodies (2 µg/mL prepared in BSA-PBS), Goat anti-Human IgG and Donkey anti-Mouse IgG, with 4 wash steps with PBS between and after antibody labeling steps to remove excess antibody solution.

**DNA barcodes and probes**

DNA barcode and probe sequences used in this work are included (**Table S1**). DNA barcodes were designed to have a unique sequence for padlock probe hybridization. Padlock probes were encoded with sequences that hybridize to fluorophore dye-linked probes that serve as a barcode for each marker of interest. For multiplexing, FAM, Cy3, and Cy5 fluorophores were attached to the probes.

**Antibody-DNA conjugation**

BSA free antibodies were buffer exchanged to PBS-bicarbonate buffer (100mM sodium bicarbonate in PBS, pH8.4) using a 40k Zeba column (LIFE Technologies, A57758). The antibody was incubated with 15 molar equivalents of TCO-PEG4-NHS Ester (Vector Labs, CCT-A137) for 25 mins at RT after which unreacted TCO-PEG4-NHS Ester was removed using two successive 40k Zeba columns. Degree of labeling (DOL) was checked by incubating antibodies with 10 molar equivalents of Cy3 Methyl Tetrazine (Vector Labs, CCT-1018) for 25 mins at RT (10% DMSO) before any remaining Cy3 Methyl Tetrazine was removed using two successive 40k Zeba columns. Cy3:Antibody ratio was measured using the Nanodrop UV/Vis mode (Thermo Scientific) at A550/A280 and calculated from the known extinction coefficients of the dye (150,000 M^−1^cm^−1^, CF280: 0.05) and protein (215,000 M^−1^cm^−1^).

1mM of amine-modified DNA oligo (Integrated DNA Technologies) was buffer exchanged to PBS-bicarbonate buffer (100mM sodium bicarbonate in PBS, pH8.4) using a 7k Zeba column (Thermo Fisher, 89878). The DNA oligo was incubated with 10 molar equivalents of Methyl Tetrazine-PEG4-NHS Ester (Vector Labs, CCT-1069) for 25 mins at RT (10% DMSO), after which excess Methyl Tetrazine-PEG4-NHS ester was cleared using three successive 7k Zeba columns. Tz:DNA ratio was calculated from Nanodrop UV/Vis measurements at A520/A260 and the known extinction coefficients of the tetrazine (438 M^−1^cm^−1^) and DNA (as supplied by the manufacturer). Measurement at two different dilutions was required given the much stronger molar absorbance of the DNA. TCO-labeled antibody and Tz-labeled DNA were mixed with appropriate DNA stoichiometry (Cy3:Antibody ratio minus 0.5, such that the TCO-antibody sites are in slight excess) and incubated for 45 mins at RT.

**Unreacted methacrylate groups characterization**

To identify the presence of remaining methacrylate groups in MHPs, 50 µL of FITC-PEG-SH (10 mg/ml) was mixed with 50 µL of MHPs in 1xPBST and incubated at 37 °C for 4 hr in a thermomixer (1500 rpm). After incubation, MHPs were washed three times in wash buffer (1xPBST). For the blocking study, 50 µL of PEG-SH (10 mg/ml) was used and reacted under the same conditions as those employed for FITC-PEG-SH. For the conjugation of protein molecules, 10 µL of cell lysate extracted from A431 cells were mixed with 90 µL of MHPs in TEOA buffer and incubated at 25 °C for 12 hr in a thermomixer (1500 rpm). After incubation, MHPs were washed three times in wash buffer (1xPBST).

**Rolling Circle Amplification assay in MHPs**

The assay is composed of four different steps: immunolabeling, ligation, amplification and fluorescent labeling. For immunolabeling, 50 µL of Ab-DNA (0.125 µg/mL) was prepared in 1xPBS with 5% BSA and mixed with 50 µL of MHPs in 1xPBST. Then, MHPs were incubated at 25 °C for 30 min in a thermomixer (1500 rpm). After incubation, MHPs were washed 3 times in wash buffer (1xPBST). For the ligation step, 50 µL of ligation mixture was prepared by combining 20 nM PLP, 400 mU/µL T4 DNA ligase (New England Biolabs, USA) in 1x T4 DNA ligase buffer (New England Biolabs). The ligation mixture was then combined with 50 µL of MHPs and incubated at 37 °C for 30 min. After three washes, 50 µL of phi29 mixture was prepared with 800 mU/µL of phi29 polymerase (Bioresearch Technologies, England), 250 µM dNTPs (New England Biolabs) in 1x phi29 DNA polymerase reaction buffer for the amplification step. The mixture was then combined with 50 µL of MHPs and incubated at 37 °C for 3 hr. Finally, after three washes, 50 µL of mixture containing 250 nM fluorescent probe in probe hybridization buffer (Molecular Instruments, USA) was combined with 50 µL of MHPs and incubated at 37 °C for 20 min. All incubation steps were conducted with a thermomixer, shaking at 1500 rpm.

**Rolling Circle Amplification assay in the microwell**

Clear scratch and UV-resistant acrylic sheets (12" x 12" x 1/8”) were used for microwell fabrication. Before laser cutting, double-sided sticky tape was placed onto one side of the acrylic for glass bondage. APLS4.75 laser system was then used to cut a 6 mm diameter hole into the acrylic sheet. After cutting, the acrylic was bonded to a cover glass slide. EVs were incubated in a microwell for 30 min at RT to settle down on the surface of the glass. After incubation, microwells were washed 3 times in wash buffer. The remaining steps follow the same protocol as those described for the RCA assay in MHPs, except for the incubation steps performed in a 37 °C incubator.

**Nano-Flow Cytometry**

As a comparison to the 2D surface-based assay, EV samples were prepared as a 3D solution-based assay with the analysis carried out using nano-flow cytometry (NanoFCM, Inc., Xiamen, China) from the University of Pennsylvania School of Veterinary Medicine Extracellular Vesicle Core. The instrument was calibrated by flowing 200 nm AF488 fluorophore-conjugated polystyrene beads for particle concentration and a Silica Nanosphere Cocktail (NanoFCM, Inc., S16M-Exo) for size distribution. To determine co-expression of CD9 and EGFR, 8 μL A431 cell line-derived EVs (1E11 particles/mL) were treated with 100 ng each of anti-EGFR-AF647 and anti-CD9-AF488 fluorophore-conjugated antibodies for at least 30 min. All samples were diluted to reach a particle count within the optimal range of 2000–12 000/min to prevent carryover of excess antibody and swarming effects. Particles that passed by the detector during a 1 min interval were recorded in each test. Dotplots were generated using the NanoFCM software (NanoFCM Profession V1.0) for analysis.

**Electron microscopy**

For the characterization of MHPs, Environmental Scanning Electron Microscopy (ESEM) was carried out using Quanta 600 FEG SEM (FEI, USA). MHPs were placed in a water containing sample holder, which was incorporated into the Peltier stage to keep the temperature at 2 °C. The chamber pressure was set to achieve 90% humidity to remove the water film from the surface layer. Accelerating voltage of 20kV was used to observe MHPs. To directly visualize EVs, Cryo Electron Microscopy was carried out by the University of Pennsylvania Perelman School of Medicine Electron Microscopy Resource Lab using a Krios G3i Cryo-TEM instrument (ThermoFisher Scientific, USA) as previously described.^[4]^ Briefly, Carbon EM grids were glow-discharged (30 s, 25 mA) in Pelco EasiGlow system, and 3 µL of PANC-1 EVs was added to the Carbon EM grid. After, samples were then blotted for 2 s and plunge-frozen into precooled liquid ethane using a Vitrobot Mark IV (FEI, USA). An accelerating voltage of 300 kV was used to observe PANC-1 EVs.

**Image acquisition and analysis**

Both MHPs and microwells were imaged using an IX83 inverted fluorescence microscope (Olympus, Japan). For imaging MHPs and immunostained cells, a 20x objective was used, while microwells required a 40x objective. The fluorescent intensity of MHPs and cells were analyzed with the aid of FIJI software (National Institute of Health, USA). The total number of dots and colocalization between dots from a different fluorescent channel was analyzed by Cell Profiler with customized pipelines specific for recognizing and counting dots. The limit of detection was calculated using 3 times standard deviation from control RCA dots. Statistical analyses were performed using GraphPad Prism version 10 (GraphPad Software Inc.). When comparing two groups, a two-tailed t-test was used. For all statistical tests, p-values < 0.05 were considered significant.


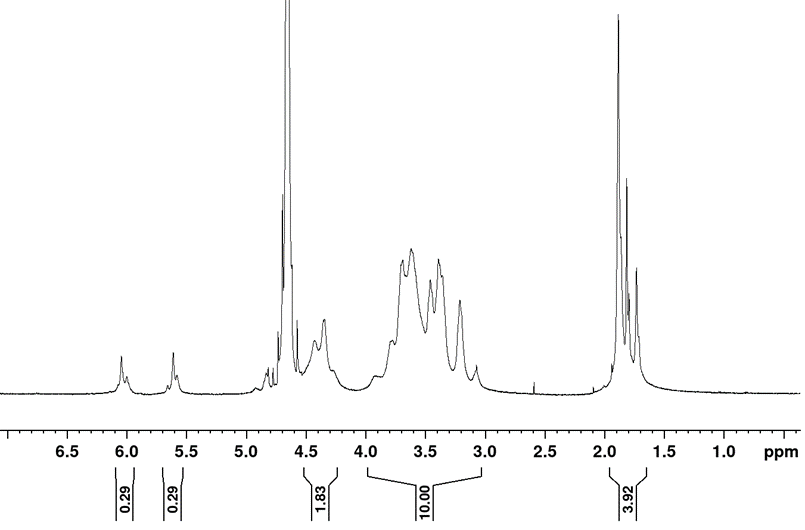


**Figure S1.** 1H NMR spectra of MeHA. Spectra were acquired at 500 MHz at a concentration of 5 mg/mL in D2O. The degree of substitution (30%) was determined by integration of the vinyl group (δ=6.05, 1 H and δ=5.66, 1 H) relative to the HA backbone (δ=3.0-4.0, 10 H).


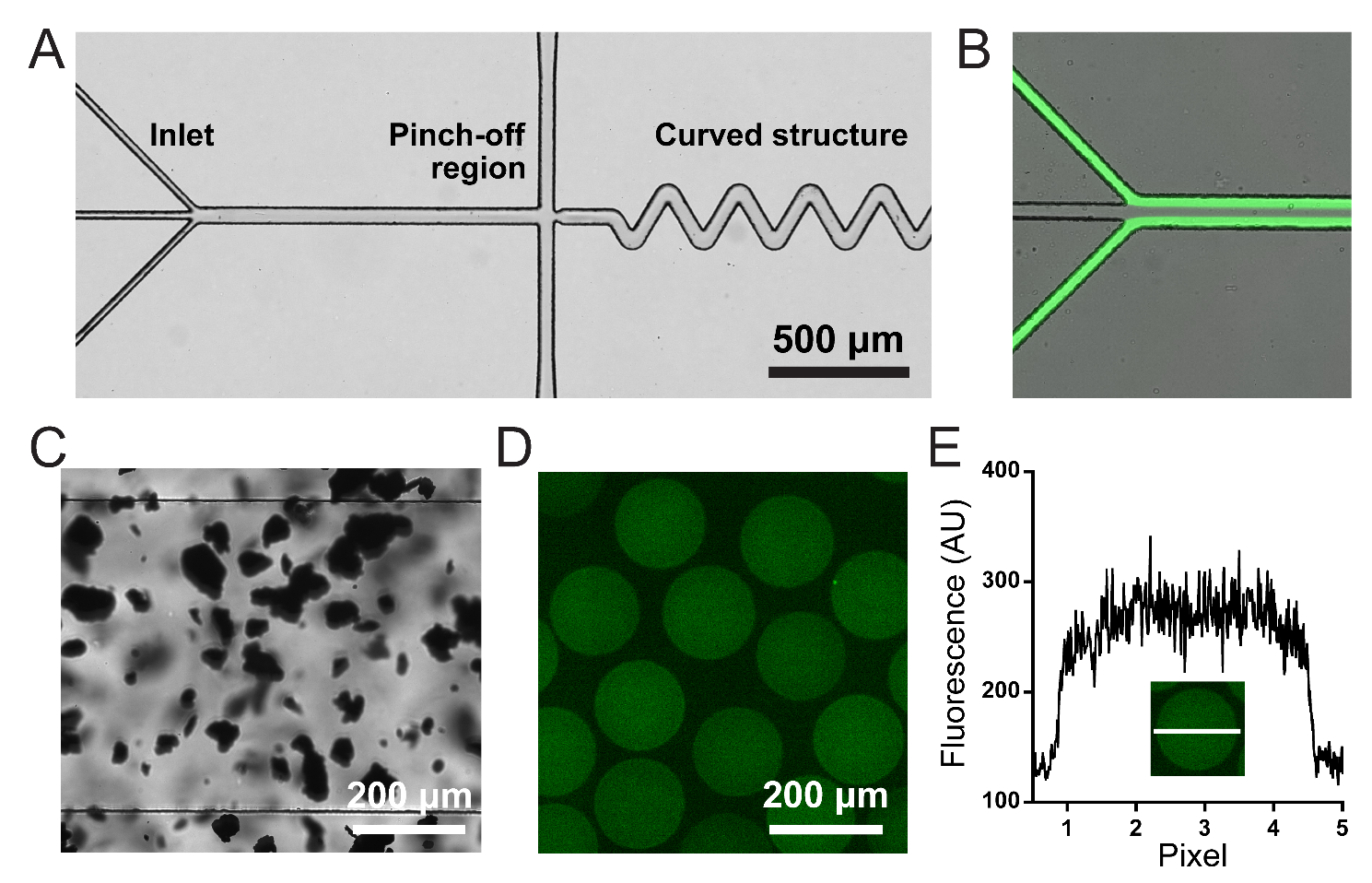


**Figure S2.** Generation of droplets. (A) A micrograph of a flow-focusing microfluidic droplet generator. (B) A merged micrograph of the inlet area. Fluorophore conjugated acrydite-DNAs were added on either side and MeHA was added in the center, mimicking the droplet generation process. (C) A micrograph of a polymerization chamber. Black dots in the micrograph are iron powder which can prevent scattering of UV inside the microfluidic device. (D) Fluorescence micrograph and (E) fluorescence plot of the hydrogels confirms the uniform mixing of the precursor components.


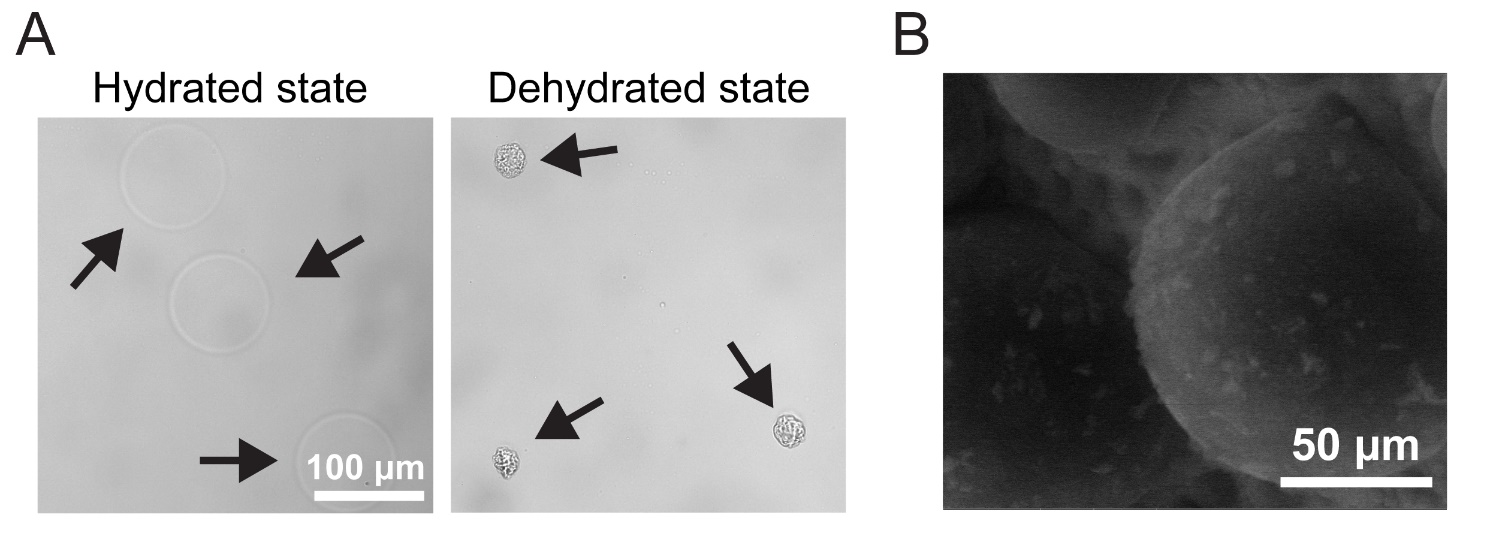


**Figure S3.** Verification of hydrogel. (A) Micrographs of MHPs in a hydrated and a dehydrated state. (B) ESEM images of MHPs. The arrows (black) point to MHPs.


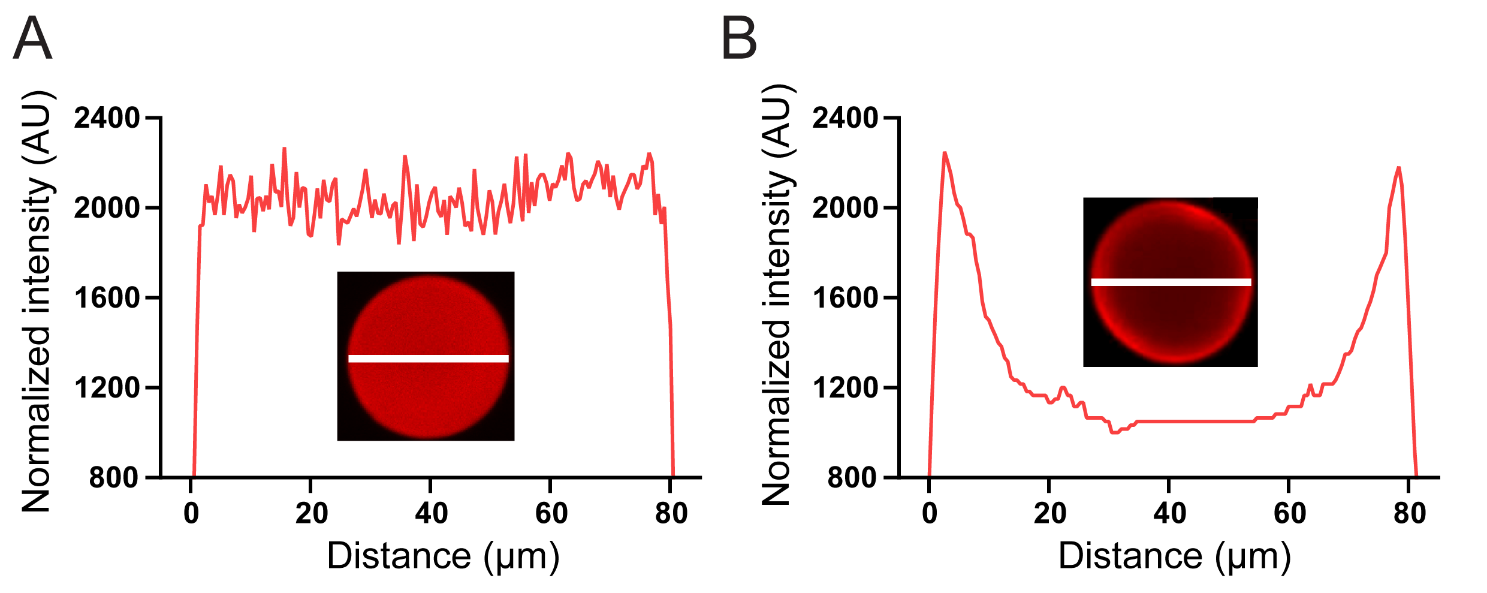


**Figure S4.** Mesh size characterization of MHPs. Normalized intensity plot of MHPs reacted with (A) fluorophore conjugated antibody and (B) fluorophore conjugated LNPs.


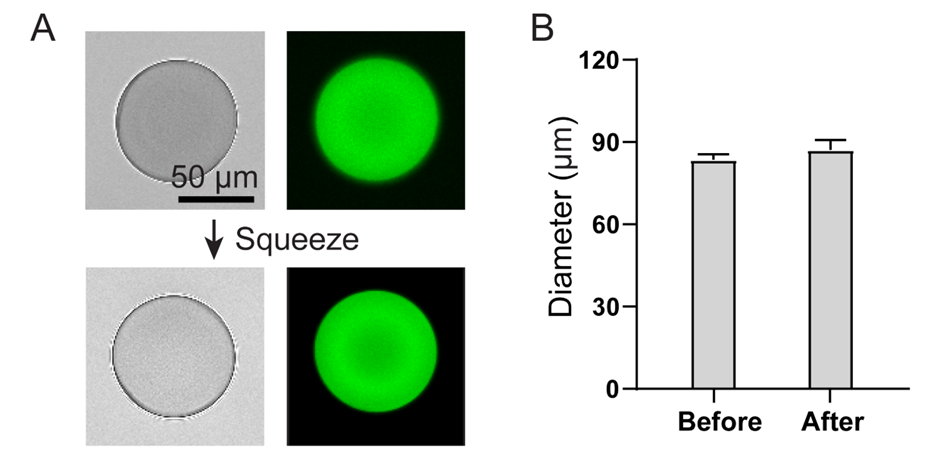


**Figure S5.** Before and after squeezing of PHPs. There was no change in diameter after squeezing PHPs. Error bars indicate the standard deviation from ten particles.

**Figure S6.** Compressibility of MHPs synthesized by different UV exposure time. Error bars indicate the standard deviation from ten MHPs. (**p < 0.01)
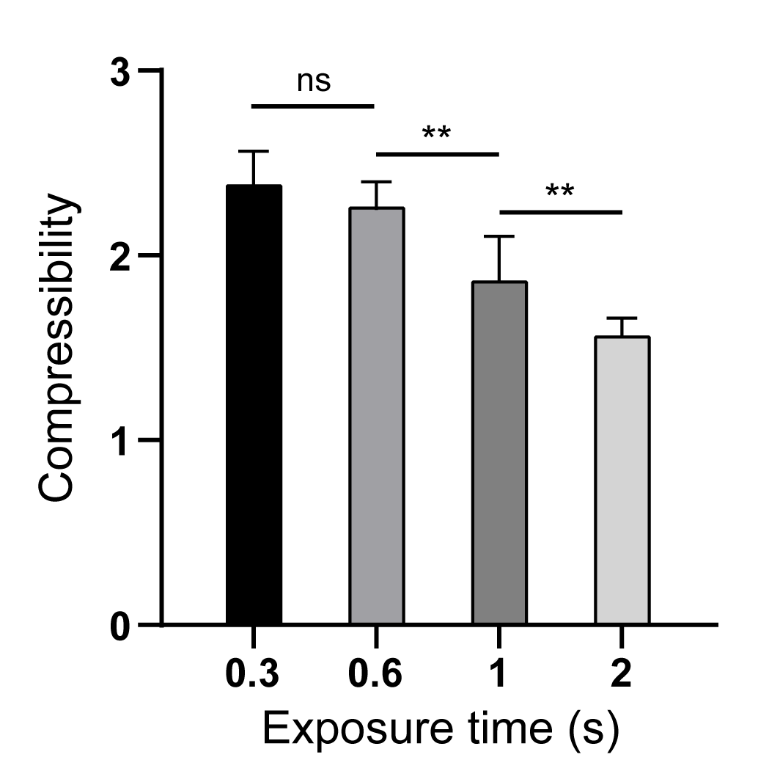


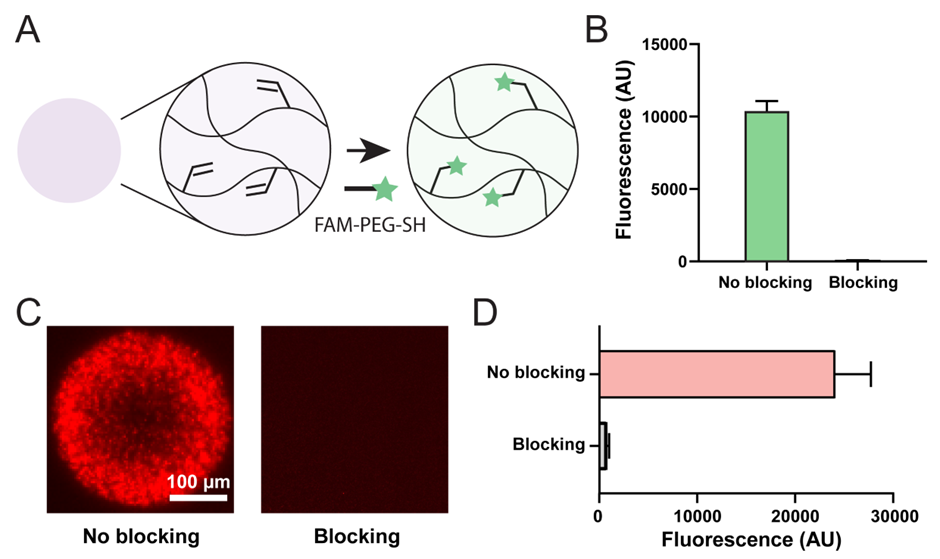


**Figure S7.** Characterization and utilization of remaining unreacted methacrylate groups in MHPs. (A) Schematic of the reaction between unreacted methacrylate groups and FAM-PEG-SH. Through thiol-ene Michael addition reaction, thiol groups are bind to electro-deficient unreacted methacrylate groups, resulting in fluorescent labeling of methacrylate groups. (B) Validation of the remaining unreacted methacrylate groups in the hydrogel. To prove that the fluorescent labeling is due to the thiol-ene reaction, one group of MHPs was treated with PEG-SH to block the unreacted methacrylate groups prior to reaction with FAM-PEG-SH. MHPs without blocking showed higher fluorescence whereas blocked MHPs showed no fluorescence. Error bars indicate the standard deviation from five MHPs. (C,D) Validation of the reaction between protein molecules and unreacted methacrylate groups. Primary amine groups of protein molecules from cell lysates form C-N bond with methacrylate groups by aza-Michael addition reaction. After reaction, RCA was performed by targeting EGFR molecules. Non-blocked MHPs showed amplified dots whereas blocked MHPs showed no dots. Error bars indicate the standard deviation from five MHPs.

**Figure S8.** Cryo-EM images of EVs isolated from PANC-1 cells. The arrows (white) point to extracellular vesicles.
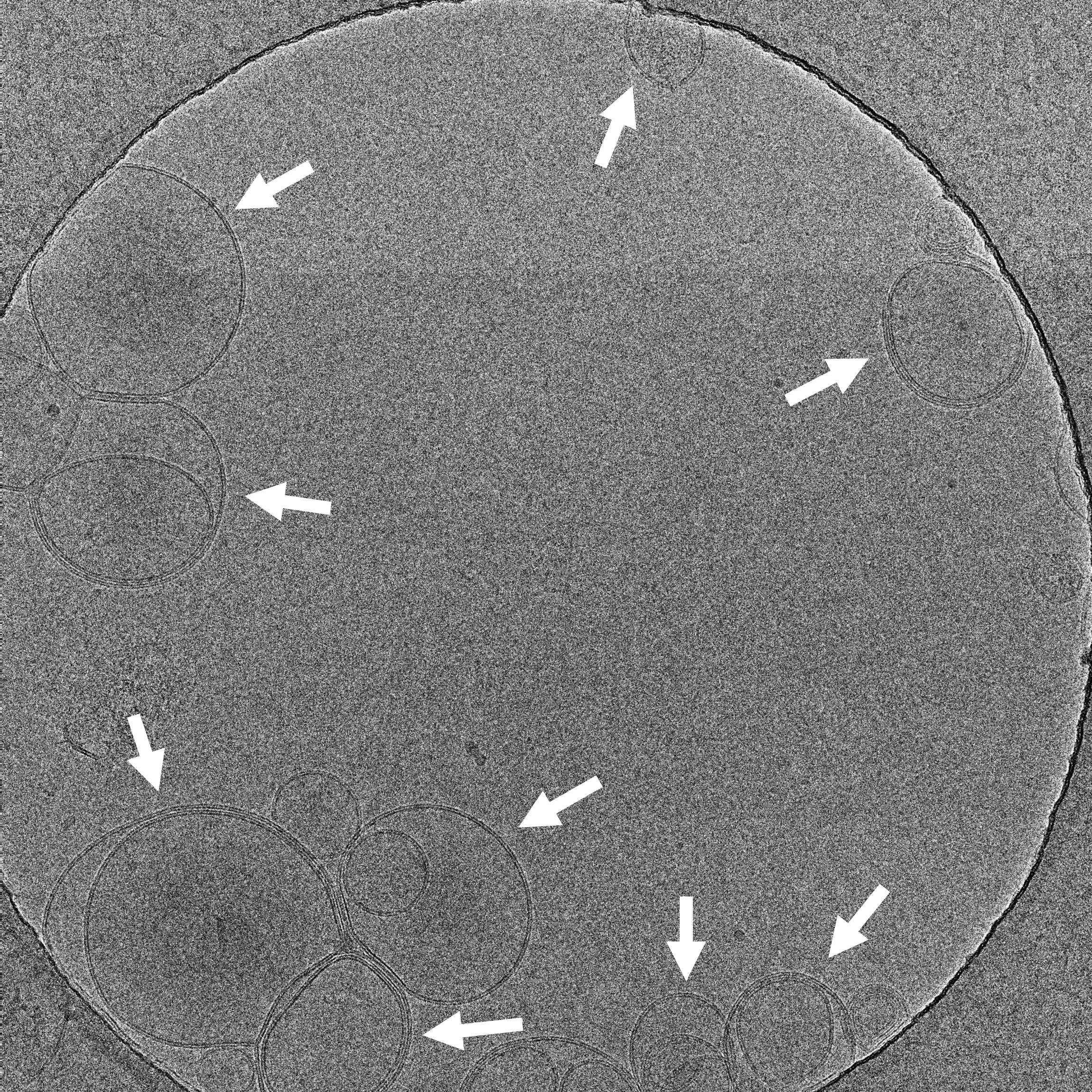


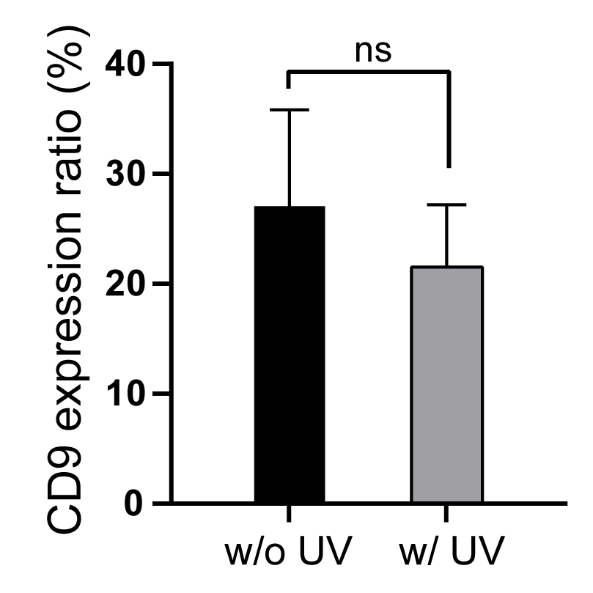
**Figure S9.** CD9 expression ratio of A431 EVs with and without UV exposure. There was no statistical difference before and after exposing UV to EVs. Error bars indicate the standard deviation from three replicates.


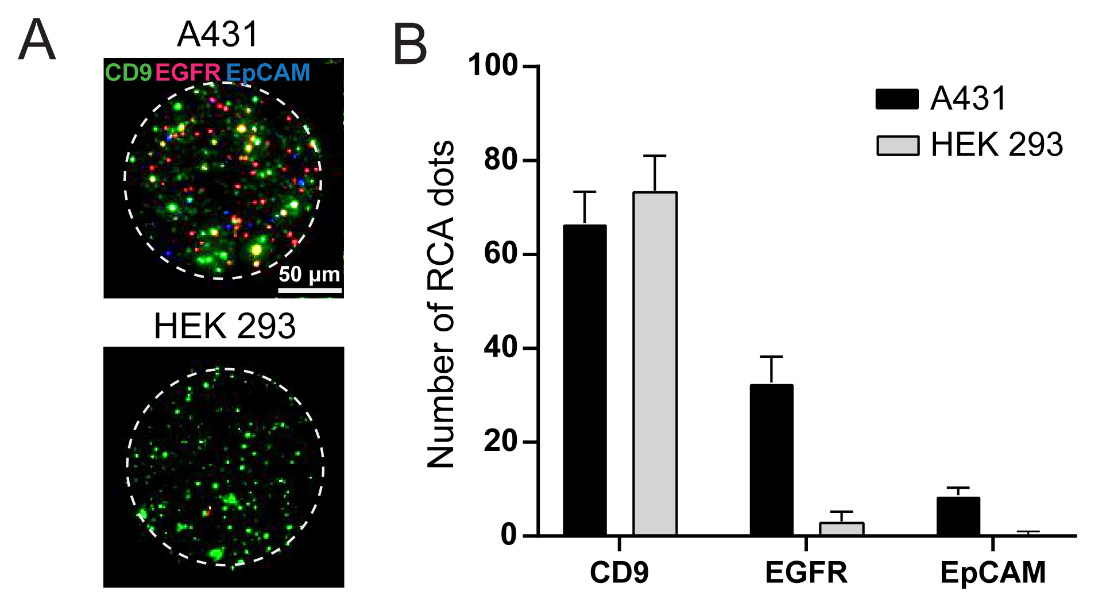
**Figure S10.** Comparison of EVs from cancerous and Non-cancerous cells. (A) Fluorescence micrographs of MHPs including A431 (cancerous cells) EVs and HEK 293 (Non-cancerous cells) EVs after 3-plex assay. (B) Bar graphs indicate number of RCA dots of CD9, EGFR and EpCAM from A431 EVs and HEK 293 EVs. Error bars indicate the standard deviation from seven MHPs.


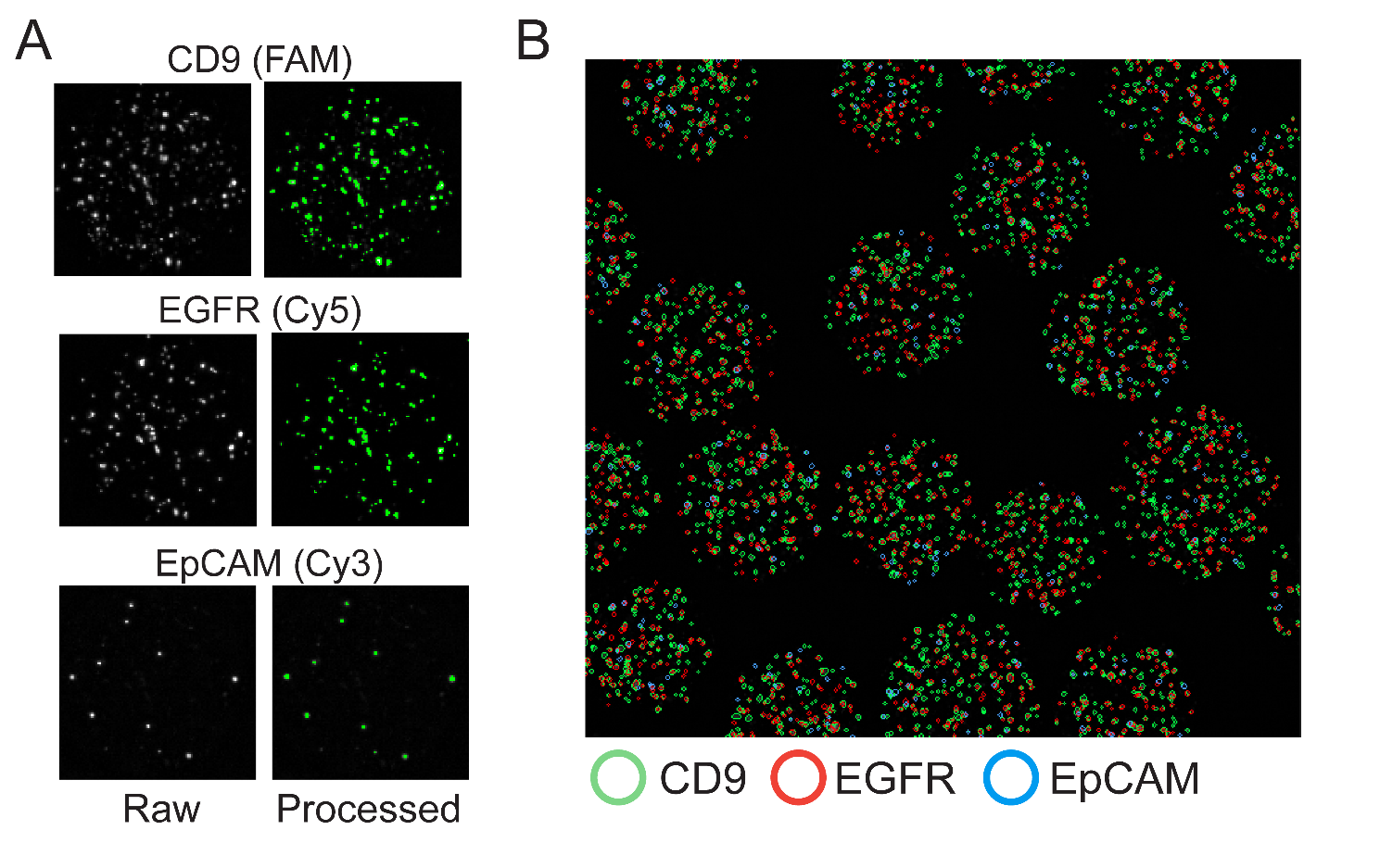


**Figure S11.** Analysis of RCA dots within the MHPs. Customized software identified the RCA dots from the fluorescence images taken from different fluorescence channels and then generated contours for counting the RCA dots. Identified contours from each fluorescence channel were combined into a single image to find the colocalization between each dot. The operating parameters of IdentifyPrimaryObjects in CellProfiler are 0.3, 0.9 for threshold smoothing scale and threshold correction factor, respectively, using Global as a threshold strategy and Otsu as a thresholding method.


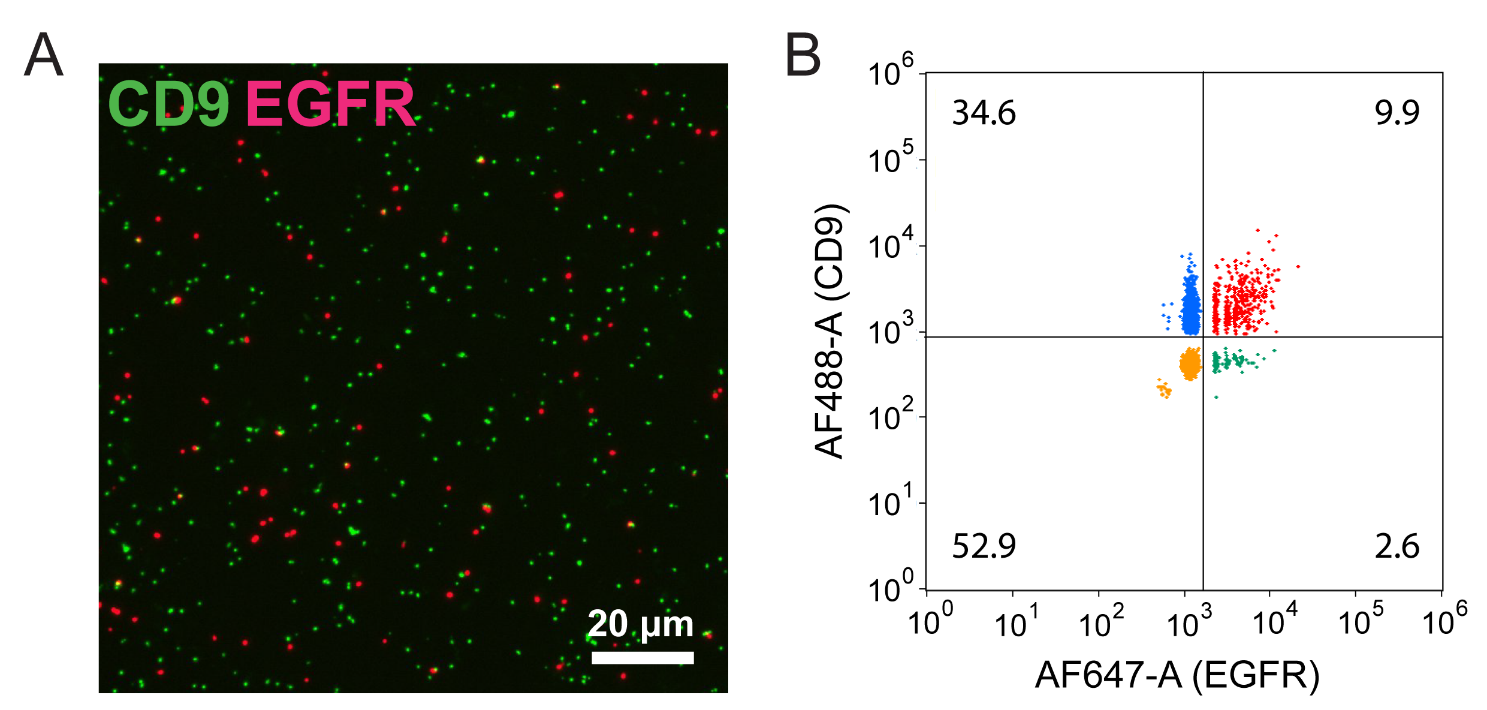


**Figure S12.** EGFR and CD9 co-expression ratio of A431 EVs analyzed by 2D surface assay and a small particle flow cytometry. (A) Fluorescence micrographs of surface after duplex RCA assay. (B) Small particle flow cytometry evaluation from 4206 gated events.


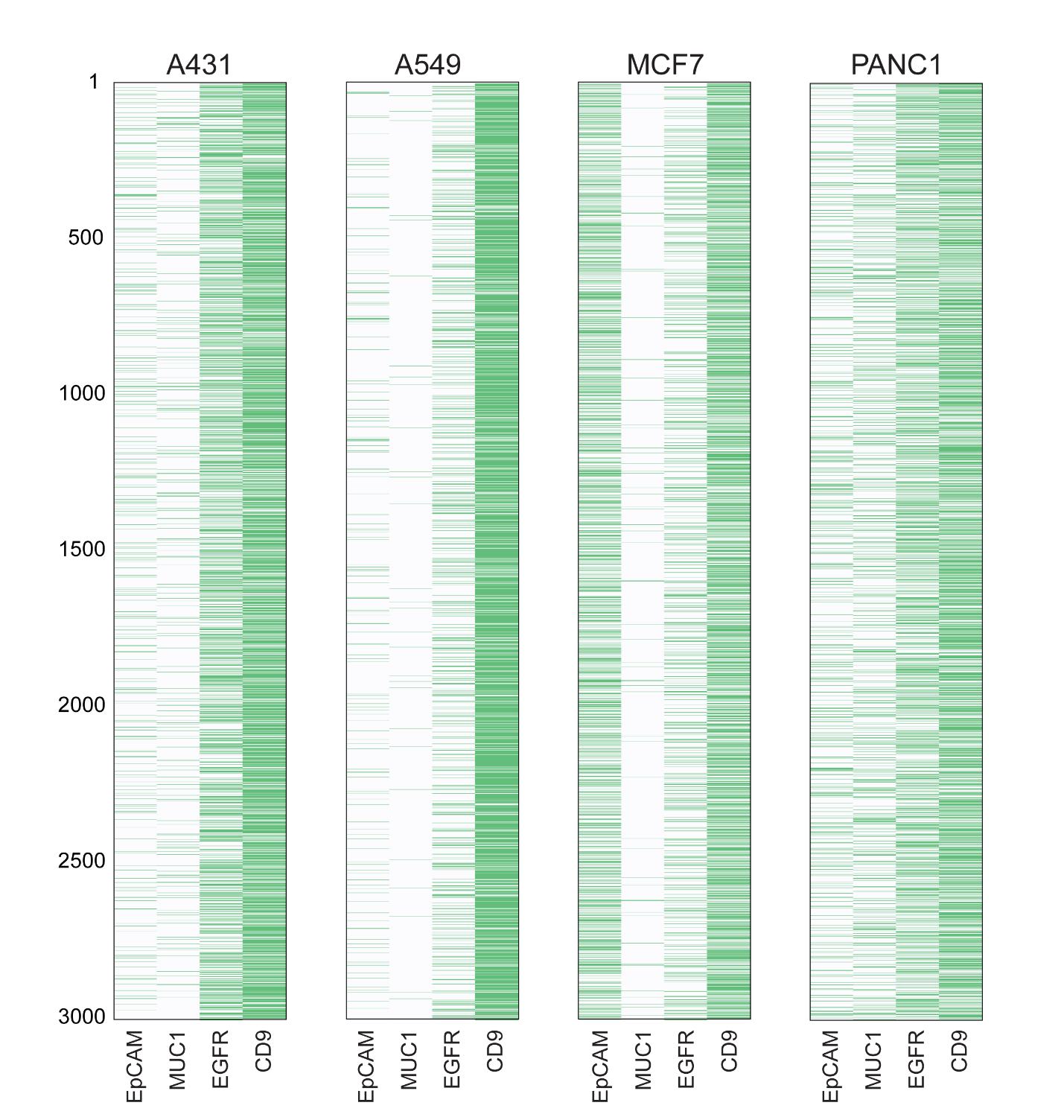


**Figure S13.** Mapping of single EV across 4 cell lines. Comprehensive single EV map was constructed across four markers from more than 4000 EVs.


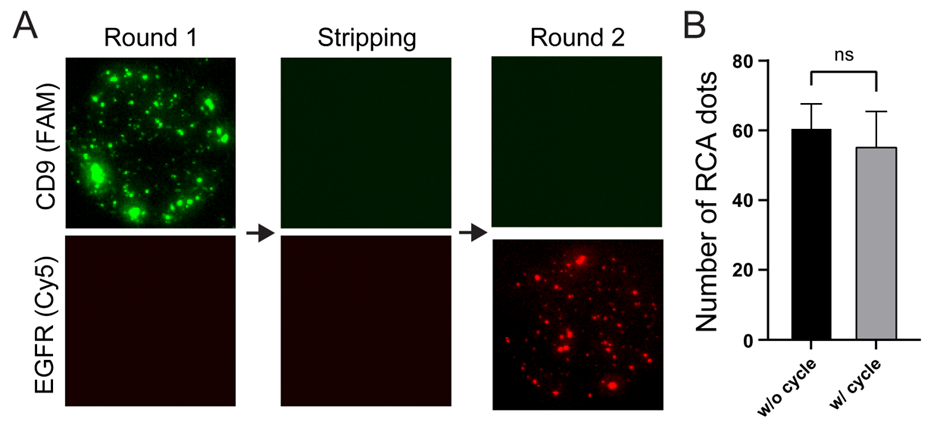


**Figure S14.** Cyclic imaging of MHPs. (A) CD9 and EGFR were targeted and amplified by RCA in A431 EV-loaded MHPs. After amplification, FAM-DNA targeting CD9 amplicons were hybridized as a first round. Then, fluorophores were stripped off by DNA denaturation using 50% formamide for 10 min. In the second round, Cy5-DNA targeting EGFR amplicons were hybridized. (B) Validation of the stability of cyclic imaging. Bars indicate the number of EGFR RCA dots with and without cyclic imaging. Error bars indicate the standard deviation from seven MHPs.

**Table S1.** DNA barcode and probe sequences

| **Component** | **DNA Sequences (5’-3’)** |
| --- | --- |
| Cy5 DNA barcode (60nt) | /5AmMC6/AAAAACCCGCCAGAAGCGATTACAGGCTCTGACTTACTAGCGTATGACGACGCGATGTTT |
| Cy5 PLP  *Arm 1, *Arm 2 (100 nt) | /5Phos/CTAGTAAGTCAGAGCCTGTATCAGAACAGTGTATGCAGCTCCTCAGTAATAGTGTCTTACAGAGAGTAGTACTTCCGACTAAACATCGCGTCGTCATACG |
| Cy5 DNA Probe (20nt) | /5Cy5/AGAGAGTAGTACTTCCGACT |
| Cy3 DNA Barcode (60 nt) | /5AmMC6/AAAAAGCTGTATGCTTCAAAGCGTTCGCATGCCTGGTTAAGTTATAACGGCAAACGTCGG |
| Cy3 PLP  *Arm 1, *Arm 2 (100 nt) | /5Phos/ATGCGAACGCTTTGAAGCATTCAGAACAGTGTATGCAGCTCCTCAGTAATAGTGTCTTACGGCTCCACTAAATAGACGCACCGTTATAACTTAACCAGGC |
| Cy3 DNA Probe (20nt) | /5Cy3/GGCTCCACTAAATAGACGCA |
| AF488 DNA Barcode (60nt) | /5AmMC6/AAAAAACGACACGTACCCCGGAGCAACGTAGATCCCGAGCATATACTGGTGTGGAGTGAA |
| AF488 PLP  *Arm 1, *Arm 2 (100 nt) | /5Phos/GCTCGGGATCTACGTTGCTCTCAGAACAGTGTATGCAGCTCCTCAGTAATAGTGTCTTACGGGCCTTATTCCGGTGCTATTTCACTCCACACCAGTATAT |
| AF488 DNA Probe (20nt) | /5AF488/GGGCCTTATTCCGGTGCTAT |
| AF 405 DNA Barcode (60nt) | /5AmMC6/AAAAACGTAATGACATATACACATGGAGTTCGGTAGCCATAACTACGCAACGCTGTCCTA |
| AF 405 PLP  *Arm 1, *Arm 2 (100 nt) | /5Phos/ATGGCTACCGAACTCCATGTTCAGAACAGTGTATGCAGCTCCTCAGTAATAGTGTCTTACCTCAATTCTGCTACTGTACTTAGGACAGCGTTGCGTAGTT |
| AF 405 DNA probe | /5AF405/CTCAATTCTGCTACTGTACT |

**References**

[1] C. Loebel, C. B. Rodell, M. H. Chen, J. A. Burdick, *Nat. Protoc.* **2017**, *12*, 1521-1541.

[2] H. J. Lee, J. Y. Kim, Y. H. Roh, S. M. Kim, K. W. Bong, *Analyst* **2019**, *144*, 6712-6720.

[3] A. P. Dhand, M. D. Davidson, J. H. Galarraga, T. H. Qazi, R. C. Locke, R. L. Mauck, J. A. Burdick, *Adv. Mater.* **2022**, *34*, 2202261.

[4] A. Emelyanov, T. Shtam, R. Kamyshinsky, L. Garaeva, N. Verlov, I. Miliukhina, A. Kudrevatykh, G. Gavrilov, Y. Zabrodskaya, S. Pchelina, *PloS one* **2020**, *15*, e0227949.
